# Erratum: RETRACTION: Efficient Quantum Transmission in Multiple-Source Networks

**DOI:** 10.1038/srep46957

**Published:** 2018-03-19

**Authors:** Ming-Xing Luo, Gang Xu, Xiu-Bo Chen, Yi-Xian Yang, Xiaojun Wang

Scientific Reports 5: Article number: 7627; 10.1038/srep07627 published online: 01 16 2015; updated: 03 19 2018.

The original version of this Article contained a typographical error in the volume number ‘5’ was incorrectly given as ‘4’. This error has now been corrected in the PDF and HTML versions of the Article.

